# *In vitro* evaluation of using ceftazidime/avibactam against carbapenem-resistant *Acinetobacter baumannii*

**DOI:** 10.1016/j.jgar.2024.06.011

**Published:** 2024-07-10

**Authors:** Nazanin Pouya, James E. Smith, Cole S. Hudson, Nicholas S. Teran, Vincent H. Tam

**Affiliations:** a Department of Pharmacy Practice and Translational Research, University of Houston College of Pharmacy, Houston, TX, USA; b Department of Pharmacological and Pharmaceutical Sciences, University of Houston College of Pharmacy, Houston, TX, USA

**Keywords:** *β*-lactam/ *β*-lactamase inhibitor, Gram-negative bacteria, Hollow fibre infection model, Pharmacokinetics, Pharmacodynamics

## Abstract

**Objective:**

Carbapenem-resistant *Acinetobacter baumannii* (CRAB) is a global concern as effective treatments are very limited. We previously used a modified susceptibility testing approach to predict growth suppression in carbapenem-resistant *Enterobacterales*, but there are uncertainties about the generalizability of the model. The objective of this study is to verify if a similar approach can be extended to CRAB.

**Method:**

A clinical isolate of CRAB resistant to ceftazidime/avibactam (CAZ/AVI, MIC = 32/4 mg/L) was examined. CAZ susceptibility was determined using increasing concentrations of AVI (0–64 mg/L), and MIC reduction was characterized with a sigmoid inhibitory maximum effect (Emax) model. The effectiveness of CAZ/AVI was validated in a hollow fibre infection model (HFIM) over 72 hours, using simulated unbound serum / epithelial lining fluid (ELF) exposures of 2.5 g over 2 hours every 8 hours. Baseline inocula of approximately 5.5 log CFU/mL were examined.

**Results:**

An AVI concentration-dependent reduction in CAZ MIC was observed (r^2^ = 0.99). CAZ MIC was dramatically reduced from 512 mg/L (no AVI) to 32 mg/L (AVI = 4 mg/L), and further to 8 mg/L (AVI = 16 mg/L). Pharmacokinetic simulations were satisfactory in the HFIM (r^2^ > 0.96). Bacterial suppression was observed > 24 hours with the serum exposure, but not that from the ELF.

**Conclusion:**

Using multiple AVI concentrations within the clinically relevant range, our susceptibility testing approach could have better insights of treatment outcome for infections caused by CRAB. This could potentially lead to effective intervention(s) overlooked by conventional susceptibility testing method. This case highlights the importance of site-specific drug exposures on determining treatment outcome.

## 1. Background

Carbapenem-resistant *Acinetobacter baumannii* (CRAB) has emerged as an escalating global health crisis. Resistance to carbapenem antibiotics, which are often considered the last resort for treating bacterial infections, has led to a challenging situation. This issue not only affects patient outcomes but also poses a significant threat to public health, with dire consequences.

Recent data collected worldwide indicated a high resistance rate of *Acinetobacter baumannii* to carbapenem antibiotics, reaching 88% in Europe [1] and 85% in Latin America [2]. Over the last twenty years, various studies have reported mortality rates for individuals with infections caused by carbapenem resistant, multidrug resistant or extensively drug resistant A. *baumannii*. These rates have ranged from 24% to 83% on a global scale. Notably, individuals with multiple pre-existing comorbidities face a significantly elevated risk of mortality when affected by a CRAB infection [3,4].

Antibiotic resistance can manifest in two ways: intrinsically, through genetic mutations within the chromosome, or acquired, through horizontal gene transfer. One prevalent form of resistance in *Enterobacterales* involves the production of *β*-lactamases, which degrade *β*-lactam antibiotics enzymatically. This enzymatic degradation represents the most frequently encountered mechanism of *β*-lactam resistance. To restore the effectiveness of *β*-lactam antibiotics, they have been combined with various *β*-lactamase inhibitors.

Traditionally, susceptibility testing of *β*-lactam/ *β*-lactamase inhibitor combinations involves determining *β*-lactam MIC using a fixed concentration of inhibitor (e.g., avibactam 4 mg/L). However, we speculate that this approach may not be adequately informative to predict treatment outcomes. Recognizing the urgency of the situation, our laboratory proposed a novel approach to optimize testing of *β*-lactam/ *β*-lactamase inhibitor combinations. In this study, we extended the modified susceptibility testing method to predict the outcomes of ceftazidime-avibactam therapy for CRAB infections.

## 2. Materials and methods

A clinical isolate of CRAB (AB FDC8) was examined. The isolate was recovered from a 24-year-old male patient with a past medical history of structural cardiovascular abnormalities and chronic kidney disease. He was hospitalized for presumed infective endocarditis, and subsequently found to have bacteremia secondary to pneumonia. The isolate was resistant to amikacin, meropenem, levofloxacin, and ceftazidime/avibactam, but genes encoding for common carbapenemases (i.e., KPC, VIM, NDM) were not detected by PCR. To ascertain drug degradation as a mechanism of resistance, a spectrophotometric assay using nitrocefin (0.5 mM in phosphate buffered saline) as the substrate was used. Bacterial isolates were grown to approximately 8 log CFU/mL in cation-adjusted Mueller Hinton broth, centrifuged at 4000x G for 15 minutes, and the pellet was resuspended in phosphate buffered saline. This bacterial suspension was frozen/thawed three times and centrifuged at 10000x G for 15 minutes. The supernatant was recovered and assayed for total protein concentration with the BCA protein assay kit (Thermo Scientific, Waltham, MA). For comparison, the phenotypic hydrolysis was performed with 0.5 μg of total cellular protein over 20 minutes at 35 °C with and without avibactam (4 mg/L).

Furthermore, ceftazidime susceptibility (MIC) was determined by the broth dilution method using increasing concentrations of avibactam (0–64 mg/L) in duplicate. Reduction in ceftazidime MIC (transformed in log2 values to reduce heteroscedasticity) was characterized mathematically with the sigmoid inhibitory maximum effect (Emax) model, using the ADAPT5 software (University of Southern California, USA). Different dynamic susceptibility (ceftazidime MIC) profiles were derived by conditioning the best-fit parameters with the target AVI pharmacokinetic profiles. The respective unbound time above instantaneous MIC (*f* %T > MIC_i_) associated with different dosing exposures were predicted by overlapping the dynamic susceptibility profiles with the target ceftazidime pharmacokinetic profiles, as illustrated previously [5]. Ceftazidime/avibactam Cmax target values for serum and ELF exposures were 100/15 mg/L and 50/6 mg/L respectively; half-life target was 2.5 hours.

Bacterial response was experimentally evaluated in the hollow fibre infection model (HFIM). Serial samples were obtained from the circulatory loop of the HFIM in duplicates to verify the simulated drug exposures. Ceftazidime and avibactam concentrations in the samples were assayed using a validated LC-MS/MS method [6]. Ertapenem was used as the internal standard. The observed concentration–time profiles were characterized using the ADAPT5 software. The simulated drug exposures would be deemed acceptable if the best-fit maximum concentrations (Cmax) and elimination half-lives are both within 20% of target values. A baseline inoculum of approximately 5.5 log CFU/mL was investigated using simulated serum/epithelial lining fluid (ELF) exposures of 2.5 g administered intravenously over 2 hours every 8 hours. Changes in bacterial density were monitored for up to 72 h by serially sampling (i.e., two independent samples were taken at each sampling) from the bioreactor cartridge. Samples were serially diluted and plated quantitatively on Mueller Hinton agar (MHA) plates. Viable colony counts were then determined following 24 h incubation at 35 °C.

## 3. Results

**Fig. 1.**
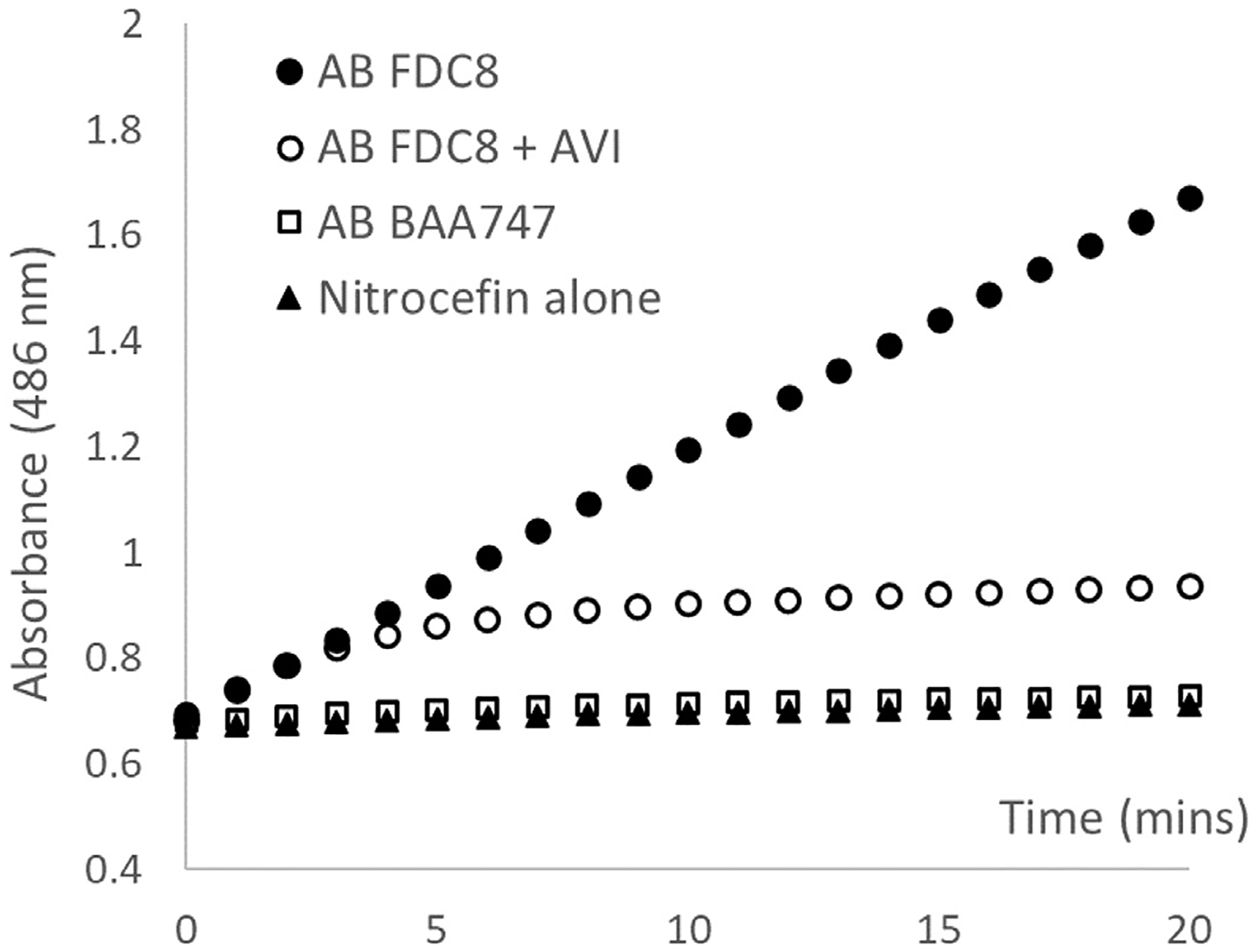
Spectrophotometric assay of nitrocefin. Note: The assay tracks the formation of hydrolysed nitrocefin product at 486 nm. AB BAA 747 is an ATCC wild-type reference (negative control); AVI (avibactam 4 mg/L).

**Fig. 2.**
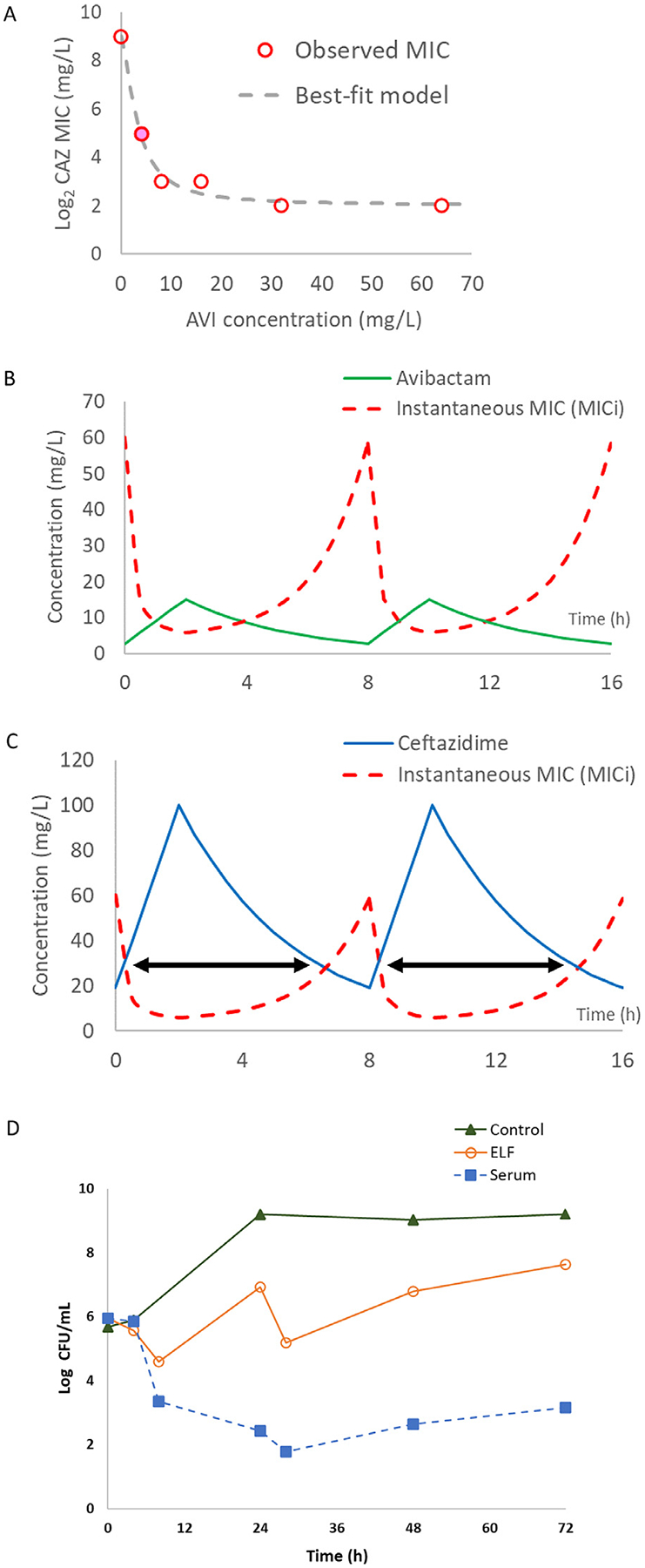
Pertinent PK/PD analysis. Ceftazidime (CAZ) MIC vs. avibactam (AVI) concentrations (A); dynamic susceptibility profile (B); profiling of *f* %T > MIC_i_ (C); experimental bacterial responses to different CAZ/AVI dosing exposures (D). Note: CAZ MIC expressed as log_2_ values. CAZ/AVI MIC = 32/4 mg/L (filled circle). Further CAZ MIC reduction was observed using AVI concentrations > 4 mg/L. Note: Instantaneous CAZ MIC (red dashed line) is inversely proportional to AVI concentrations (green line) in our modelling framework. A high AVI concentration (shortly after dosing—around 2 h) is associated with a low CAZ MIC value, whereas a low AVI concentration (towards the end of a dosing interval—around 8h) is associated with a high CAZ MIC value. Serum AVI concentration-time profile shown. Note: The *f* %T > MIC_i_ associated with a dosing exposure (black arrows) is derived by overlapping the dynamic susceptibility profiles (red dashed line) with a CAZ pharmacokinetic profile (blue line). Serum CAZ concentration-time profile shown.

Despite the absence of common carbapenemase genes, enzymatic activity of crude bacterial cell lysate (i.e., nitrocefin hydrolysis) was observed. The hydrolysis was partially inhibited by the addition of avibactam, as shown in Fig. 1. An AVI concentration-dependent reduction in ceftazidime MIC was well characterized (r^2^ = 0.99), as shown in Fig. 2A. Notably, the MIC of ceftazidime decreased significantly from 512 mg/L (when no AVI was present) to 32 mg/L (with AVI at 4 mg/L), and further to 8 mg/L (when AVI reached 16 mg/L). Using a novel concept of dynamic susceptibility (Fig. 2B), the corresponding *f* %T > MIC_i_ was predicted to be 80.0% for serum drug exposures (Fig. 2C) and 30.8% for ELF exposures, respectively. Based on the conventional method (i.e., in reference to a fixed CAZ/AVI MIC of 32/4 mg/L), the *f* %T > MIC would be 72.3% (serum) and 31.3% (ELF), respectively.

Pharmacokinetic simulations in the HFIM were satisfactory (r^2^ > 0.96) (Data not shown). Bacterial responses to simulated ceftazidime/avibactam exposures are shown in Fig. 2D. Bacterial growth suppression was observed for longer than 24 hours with the serum drug exposures as predicted. A consistent trend was observed with experiments performed on different days (data not shown). In contrast, regrowth was observed with the ELF drug exposures.

## 4. Discussion

CRAB is a challenging problem in healthcare. It displays high resistance to first-line antibiotics, severely restricting effective treatment options. The current approach involves utilizing molecular methods to detect the presence of specific resistance genes. However, the presence of a gene does not always correlate to functional expression of enzyme(s). As we have shown previously, inference to MIC elevation may also not be straightforward if there are multiple resistance determinants involved [7].

The conventional method for susceptibility testing of *β*-lactam/ *β*-lactamase inhibitor combinations often employ a single concentration of the inhibitors, which may not fully represent the range of concentrations encountered in the body. This restricted approach could potentially lead to inaccurate assessment of bacterial response to treatment. To circumvent these limitations, our approach combines elements of quantitative analysis by employing a range of concentrations within the clinically relevant range. This method is also relatively easy to implement in a clinical microbiology laboratory, without acquiring expensive equipment for molecular-based investigations.

We have previously demonstrated this approach in *Enterobacterales*. By utilizing multiple concentrations of the *β*-lactamase inhibitor (tazobactam or avibactam), our susceptibility testing method refines the assessment, providing more comprehensive insights of bacterial response to therapy. This approach could potentially lead to the identification of effective intervention(s) that might be overlooked by conventional susceptibility testing methods. Ultimately, this method aims to optimize patient care by tailoring treatment for improved efficacy and addressing the pressing issue of antibiotic resistance in a more targeted manner.

In this anecdotal case report, we explored if a more accurate prediction of treatment outcomes for CRAB infections is feasible, without having comprehensive knowledge of the resistance mechanisms involved [6]. Notably, ceftazidime/avibactam is not routinely recommended against CRAB. Despite the isolate being classified as resistant by conventional testing methods, the projected PK/PD exposure in serum marginally surpassed the previously reported target threshold (*f* %T > MIC_i_ ≥ 76.1%) [6]. This highlights a potential discrepancy between conventional classifications of resistance and the actual performance observed under dynamic drug exposures, emphasising the need for more robust susceptibility testing approaches. Given the bloodstream infection of the patient is suspected to be secondary to pneumonia, we recognize therapy with ceftazidime/avibactam is unlikely to be effective. Nonetheless, we have predicted bacterial responses using serum and ELF drug exposures reasonably well in a pre-clinical infection model, further attesting the utility of our approach.

Several limitations should be recognized. First, the study was limited to only one CRAB isolate exposed to ceftazidime/avibactam. Additional resistant isolates should be examined in future studies to ascertain if there is a robust threshold exposure to predict bacterial suppression, and also for other *β*-lactam/ *β*-lactamase inhibitor combinations. For optimal clinical application, the anticipated drug exposures at the site of infection (i.e., source of bacteremia) must be known. Moreover, *in vitro* findings of this study should be validated in an animal infection model.

In conclusion, therapy efficacy might be better explained by MIC profiling of ceftazidime/avibactam. This method appears to be more robust than conventional susceptibility testing, and the clinical utility of this approach should be further investigated.
